# Guideline-Concordant Care and Clinician and Clinic Characteristics for Patients With Schizophrenia

**DOI:** 10.1001/jamanetworkopen.2025.49130

**Published:** 2025-12-26

**Authors:** Annie Yu-An Chen, Gregory E. Simon, Keith M. Ericson, John E. Zeber, Jing Qian, Kimberley H. Geissler

**Affiliations:** 1 Department of Healthcare Delivery and Population Sciences, University of Massachusetts Chan Medical School–Baystate, Springfield; 2 Kaiser Permanente Washington Health Research Institute, Seattle; 3 National Bureau for Economic Research, Cambridge, Massachusetts; 4 Boston University Questrom School of Business, Boston, Massachusetts; 5 School of Public Health and Health Sciences, University of Massachusetts, Amherst

## Abstract

**Importance:**

Guideline-concordant care of schizophrenia remains low despite available evidence-based treatments. Primary care providers (PCPs; primary care clinicians) and psychiatric advanced practice practitioners (APPs) increasingly deliver specialty mental health care, but whether care quality differs by clinician or clinic type or patient mix is unclear.

**Objective:**

To assess whether clinician or clinic specialty and higher schizophrenia caseload intensity are associated with receipt of guideline-concordant schizophrenia care.

**Design, Setting, and Participants:**

This cross-sectional study (January 1, 2014, to December 31, 2021) used multivariable logistic regression to assess associations between clinician or clinic specialty and guideline-concordant care, adjusting for patient and zip code characteristics. Some models included interactions between specialty and a high schizophrenia caseload intensity indicator to examine associations within specialties. Insured individuals aged 18 to 64 years with schizophrenia from the Massachusetts All Payer Claims Database were studied. Analysis was completed between August 1, 2024, and September 25, 2025.

**Exposures:**

Clinician or clinic specialty and schizophrenia caseload intensity.

**Main Outcomes and Measures:**

Binary indicators of high medication adherence, any receipt of psychosocial services, routine receipt of psychotherapy, high inpatient use, and receipt of antipsychotic-related diabetes screening.

**Results:**

The final analytic sample included 29 713 person-year observations (18 772 [63.2%] male; 5569 [63.2%] aged 35-64 years). Among these, 12 104 (40.7%) were attributed to psychiatrists, 3144 (10.6%) to psychiatric APPs, 6626 (22.3%) to mental health clinics, and 7839 (26.4%) to PCPs. In adjusted regressions, patients of psychiatric APPs or mental health clinics had higher probabilities of receiving psychosocial services (APP vs psychiatrist: 80.1% vs 72.1%, *P* < .001; mental health clinic vs psychiatrist: 87.1% vs 72.1%, *P* < .001) and routine psychotherapy (APP vs psychiatrist: 30.4% vs 24.0%, *P* < .001; mental health clinic vs psychiatrist: 35.2% vs 24.0%, *P* < .001) than patients of psychiatrists; antipsychotic adherence was similar or higher. In contrast, those with PCPs were less likely to receive psychosocial services (–15.85 percentage points [pp]; 95% CI, –18.40 to –13.30 pp) or routine psychotherapy (–16.42 pp; 95% CI, –17.80 to –15.05 pp) but more likely to receive antipsychotic-related diabetes screening (5.66 pp; 95% CI, 4.02-7.30 pp). High inpatient use did not significantly differ. Attribution to PCPs with higher schizophrenia caseload intensity was not consistently associated with improved outcomes across specialties.

**Conclusions and Relevance:**

In this cross-sectional study, guideline-concordant care was similar for patients of psychiatric APPs, mental health clinics, and psychiatrists, but patients of PCPs were less likely to receive psychosocial or psychotherapy services, although higher schizophrenia caseload intensity may mitigate this gap. Strategies to improve care across specialties are needed because overall rates remain low.

## Key Points

**Question:**

Does receipt of guideline-concordant care for patients with schizophrenia differ by clinician or clinic type?

**Findings:**

In this cross-sectional study, patients with schizophrenia treated by individual psychiatric advanced practice practitioners and mental health clinics had guideline-concordant care rates comparable to or better than those treated by individual psychiatrists. Guideline-concordant care rates for those treated by primary care clinicians or community mental health clinics were mixed compared with rates for those treated by psychiatrists.

**Meaning:**

This study suggests that advanced practice practitioners provide similar care, whereas those receiving care from primary care clinicians or community mental health clinics may require additional attention to ensure appropriate care receipt.

## Teaser

This cross-sectional study of patients with schizophrenia examines whether those receiving care from a psychiatrist have higher rates of guideline-concordant care than those receiving care from primary care clinicians, mental health clinics, or advanced practice practitioners.

## Introduction

Schizophrenia is associated with high rates of adverse health and social outcomes, including premature mortality.^1,2^ Evidence-based treatment can help manage symptoms and improve quality of life. The American Psychiatric Association (APA) practice guidelines for schizophrenia treatment recommend a combination of pharmacotherapy, psychosocial interventions, and preventive care.^3^ Research suggests psychosocial services and psychotherapy reduce psychotic relapses in individuals with schizophrenia,^4^ whereas adherence to antipsychotic medication can also reduce relapses and decrease the need for acute care services, such as hospitalizations and emergency department (ED) visits.^5,6,7,8^ Despite this evidence, rates of guideline-concordant care, including psychosocial services, psychotherapy, and antipsychotic medication adherence, remain low among people with schizophrenia.^9,10^

The care burden of schizophrenia is further exacerbated by systemic health care coverage limitations and access to specialty care.^11,12^ In the US, Medicaid is the primary payer for mental health services,^13^ covering nearly 70% of individuals with schizophrenia after the Patient Protection and Affordable Care Act.^11^ However, fewer than half of psychiatrists accept Medicaid patients.^14^ Low practitioner participation restricts care access for adults with serious mental illness, leading to disproportionately high clinical caseloads for participating practitioners and prolonged wait times for patients.^15^

In recent years, one strategy to improve access to mental health specialty care is expanding the role of primary care providers (PCPs; primary care clinicians), along with advanced practice practitioners (APPs), including physician assistants (PAs) and nurse practitioners (NPs), with psychiatric training. Community health centers, similar to community mental health clinics, are often staffed with a higher share of APPs compared with a physician-focused staffing model.^16^ During the past few decades, the proportion of patients with mental disorders has increased in primary care settings, and antipsychotic prescribing has increased more rapidly in primary care than in psychiatric settings.^15,17^ APPs have also played an increasing role in delivering specialty mental health care, with the number of residency matches for psychiatric mental health nurse practitioners increasing from fewer than 1000 in 2013 to 7000 in 2023.^18^

Despite these shifts, limited research has examined the quality of schizophrenia treatment by PCPs or APPs compared with psychiatrists. A study^19^ using data from the 1992 National Comorbidity Survey found that patients with serious mental illness were more likely to receive evidence-based treatment and at least minimally adequate care when seen by mental health specialists compared with those treated in the general medical sector by nonpsychiatrist physicians. The authors suggested that care disparities may be driven by insufficient training among general medical practitioners as well as competing demands PCPs face for time and resources. As efforts to expand access to mental health specialty care increasingly involve PCPs and APPs, further research is needed to determine whether rates of guideline-concordant care vary by clinician or clinic specialty or patient panel composition in recent years.

Using statewide data from 2014 to 2021, we examined associations between clinician or clini specialty and receipt of guideline-concordant care among individuals with schizophrenia as well as the association of high schizophrenia caseload intensity with outcomes for each specialty. We hypothesized that those receiving care from a psychiatrist will have higher rates of guideline-concordant care than those receiving care from PCPs, mental health clinics, or APPs. We also hypothesized that patients of clinician or clinics with high schizophrenia caseload intensity would be associated with higher rates of guideline-concordant care for all specialties.

## Methods

### Data

In this cross-sectional study, we used the 2014 to 2021 Massachusetts All-Payer Claims Database, which includes insurance eligibility data and medical and prescription claims from public and private insurers in Massachusetts.^20^ Our study population consisted of individuals aged 18 to 64 years with schizophrenia with at least 7 months of primary medical insurance in a year between January 1, 2014, and December 31, 2021; observations are at the person-year level. We required at least 1 hospital visit (inpatient, ED, or observation) or 2 outpatient visits with a principal or secondary diagnosis of schizophrenia (*International Classification of Diseases, Ninth Revision (ICD-9) *code 295.X excluding 295.7; *International Statistical Classification of Diseases and Related Health Problems, Tenth Revision (ICD-10)* code F20.X). We included observations with insurance types of Medicaid, private insurance, Medicare, or Health Safety Net. Of note, because Medicare fee-for-service data are not included, Medicare only includes Medicare Advantage and the integrated Medicare-Medicaid plan in Massachusetts. In Massachusetts, uninsured or underinsured residents can enroll in the Health Safety Net for certain services at designated hospitals and health centers, with enrollment and use tracked in the data. This study followed the Strengthening the Reporting of Observational Studies in Epidemiology (STROBE) reporting guidelines^37^ and was reviewed and deemed exempt by the Baystate Medical Center Institutional Review Board because the study used secondary analysis of existing data.

### Variables

As outcomes, we chose guideline-concordant care indicators based on APA’s practice guideline for schizophrenia treatment and past literature, focusing on indicators reliably measured in claims data containing multiple insurance types.^3,9,21^ The primary outcomes include high medication adherence for antipsychotic medications (defined as proportion of days covered [PDC] ≥0.8; PDC calculated using the number of days with medication divided by the number of insured days), any receipt of psychosocial services, routine receipt of psychotherapy, and receipt of diabetes screening for individuals taking antipsychotic medications. Additionally, we examined high use of inpatient care for schizophrenia (defined as ≥30 inpatient days or ≥3 inpatient admissions with a principal diagnosis of schizophrenia) as an indicator of less effective care, acknowledging this may also be an indicator of more severe disease. Observations were classified as using antipsychotic medication if they had at least one prescription for an antipsychotic drug within a calendar year, identified through prescription claims. Outcomes were determined using *Current Procedural Terminology* (*CPT*) codes and Healthcare Common Procedure Coding System codes from medical claims (see eTable 1 in Supplement 1 for full definitions).^22,23,24,25,26,27,28^

The key independent variables were the attributed clinician or clinic specialty and their schizophrenia caseload intensity. We identified National Provider Identifiers (NPIs) from medical claims and used data from the National Plan and Provider Enumeration System and National Uniform Claim Committee to determine the specialty of each NPI.^29,30^ We attributed observations to clinicians or clinics based on the number of mental health medication management or outpatient evaluation and management visits (collectively referred to as outpatient visits [*CPT* codes 90862, 90805, 90807, 90809, 99202-99215]). We used a hierarchical attribution method to best capture the clinician or clinic leading care for schizophrenia, attributing observations in the following order if there was at least one outpatient visit with a clinician or clinic in that category: (1) psychiatrists; (2) PAs or NPs with psychiatric specialty; (3) registered nurse (RN) with psychiatric specialty; (4) mental health clinics; (5) other behavioral health clinicians (including neurologists, counselors, and social workers); and (6) PCPs, including physicians and APPs (eTable 2 in Supplement 1). Some observations were attributed to mental health clinics, many of which do not bill for specific clinicians; we included this as a category because these observations are receiving mental health specialty care but were not attributed to an individual NPI in 1 of the first 3 categories. After using hierarchical attribution, we eliminated patients attributed to categories 3 (RNs with psychiatric specialty) and 5 (other behavioral health practitioners) because they are generally not responsible for prescribing or managing antipsychotic medications. If an observation has outpatient visits with more than one clinician or clinic in a category, we assign it to the modal clinician or clinic in that category.

Schizophrenia caseload intensity was measured at the NPI-year level by calculating the proportion of outpatient visits with any schizophrenia diagnosis (outpatient visits with primary or secondary diagnosis of schizophrenia divided by all outpatient visits in year). Observations were classified as having a clinician or clinic with high schizophrenia caseload intensity if the attributed clinician’s or clinic’s proportion of such outpatient visits met or exceeded the 75th percentile for the same specialty in the analytic dataset.

Other variables include age category, sex, primary insurance type, comorbidities, and index of neighborhood-level resources at the 5-digit zip code level.^31^ Primary insurance type is assigned as the modal primary insurance type in the year. The index of neighborhood-level resources is a continuous variable capturing area-level resources across multiple domains, including the built environment, criminal justice, education, employment, housing, income and poverty, social cohesion, transportation, and wealth^31^; although not an ideal proxy for individual-level resources,^32^ area-level resources are associated with health outcomes.^31,33,34^ We used indicators for Elixhauser comorbidities, excluding alcohol or drug abuse (due to data availability) and psychosis.^35^ Those with missing data on any included variable were excluded (eFigure 1 in Supplement 1).

### Statistical Analysis

First, multivariable logistic regressions were used to estimate the associations of specialty with outcomes of interest, controlling for age category, sex, primary insurance type, comorbidities, an index of neighborhood-level resources at the 5-digit zip code level,^31^ fixed effects for 3-digit zip code of residence, and calendar year fixed effects. In a second set of models, the attributed clinician or clinic specialty is interacted with a binary indicator for high schizophrenia caseload intensity to assess how a high proportion of patients with schizophrenia in a clinician’s or clinic’s panel relates to outcomes within each specialty. In comparisons among specialties, those cared for by psychiatrists are used as the reference category because the central policy question is whether the quality of schizophrenia management differs between psychiatrists and other clinician types. We tested statistical differences using Wald tests from logistic regression between each specialty and psychiatry and between high and low schizophrenia caseload intensity within each specialty.

Because the outcomes have different base rates and the multivariable logistic regression model includes interaction terms, interpreting the adjusted odds ratios for specialty and high schizophrenia caseload intensity is not straightforward.^36^ We therefore focus on regression-adjusted predicted probabilities and average marginal effects, which are more clinically meaningful. Regression-adjusted predicted probabilities in the first set of models were estimated by assigning all observations to a given specialty while holding all other covariates at their observed values. In the second set, we used a similar method, estimating for each value of schizophrenia caseload intensity within specialty. Average marginal effects are calculated to show differences between predicted probabilities; we note in the text any differences in significance vs these average marginal effects. To ensure our results were not driven by differences in service availability during the COVID-19 pandemic, we conducted a sensitivity analysis eliminating observations from 2020 and 2021.

Statistical significance was defined as a 2-sided *P* < .05, with all tests being 2-tailed. Robust SEs were clustered at 5-digit zip code level. SEs for predicted probabilities and average marginal effects were calculated using the Δ method. Data management and analyses were conducted using Stata-MP software, version 18.5 (StataCorp) and SAS, version 9.4 (SAS Institute Inc). Analysis was completed between August 1, 2024, and September 25, 2025.

## Results

The final analytic sample included 29 713 person-year observations. Of the observations, 18 772 (63.2%) were male and 10 946 (19.4%) were female. Most were between the ages of 35 and 64 years (5569 [63.2%]). Beginning with the 56 510 observations with at least 1 ED visit or 1 hospitalization or 2 outpatient visits with a schizophrenia diagnosis, 5690 (19.1%) did not have any qualifying outpatient visits needed to attribute them to an outpatient clinician or clinic (eFigure 1 in Supplement 1). Those without a qualifying outpatient visit were more likely to be younger, male, insured under Medicaid, and residing in neighborhoods with worse resources and less likely to have any comorbidities (eTable 3 in Supplement 1). An additional 28% were excluded because they lacked an attributed clinician or clinic with a specialty of interest; most of these were attributed to other specialists such as neurologists or to group NPIs for which we could not determine a specialty.

In our analytic sample, 12 104 (40.7%) included at least one qualifying outpatient visit with a psychiatrist (Table 1). Physician assistants (PAs) and nurse practitioners (NPs) specializing in psychiatry served as the attributed clinician or clinic for 3144 observations (10.6%), whereas mental health clinics provided care for 6626 (22.3%) and PCPs for 7839 (26.4%). Our sample was predominantly male and Medicaid insured, and 24 638 (83.1%) had at least one comorbidity. The most common comorbidities were depression, hypertension, and obesity. Differences in characteristics were observed by clinician or clinic specialty. Most notably, those under the care of PCPs were older (26.9% in 55- to 64-year category for PCP vs 23.0% for psychiatrists, *P* < .001 for age distribution) and more likely to be Medicaid insured (80.2% for PCP vs 56.6% for psychiatrist, *P *< .001) than those cared for by psychiatrists.

**Table 1.** Descriptive Statistics of Study Population by Attributed Clinician or Clinic Specialty^a^

Characteristic	No. (%) of person-years^b^				
	Overall (N = 29 713)	Attributed clinician or clinic specialty			
		Psychiatrist (n = 12 104)	PA or NP with psychiatric specialty (n = 3144)	Mental health clinic (n = 6626)	PCP (n = 7839)
Age group, y					
18-24	3979 (13.4)	1762 (14.6)	426 (13.5)	897 (13.5)	894 (11.4)
25-34	6962 (23.4)	2900 (24.0)	724 (23.0)	1648 (24.9)	1690 (21.6)
35-44	5475 (18.4)	2165 (17.9)	582 (18.5)	1289 (19.5)	1439 (18.4)
45-54	6162 (20.7)	2499 (20.6)	643 (20.5)	1315 (19.8)	1705 (21.8)
55-64	7135 (24.0)	2778 (23.0)	769 (24.5)	1477 (22.3)	2111 (26.9)
Sex					
Male	18 696 (62.9)	7479 (61.8)	1993 (63.4)	4409 (66.5)	4815 (61.4)
Female	11 017 (37.1)	4625 (38.2)	1151 (36.6)	2217 (33.5)	3024 (38.6)
Insurance type					
Medicaid	19 873 (66.9)	6849 (56.6)	1669 (53.1)	5071 (76.5)	6284 (80.2)
Private	5393 (18.2)	3170 (26.2)	669 (21.3)	671 (10.1)	883 (11.3)
Medicare Advantage and integrated Medicare and Medicaid	3130 (10.5)	1386 (11.5)	543 (17.3)	690 (10.4)	511 (6.5)
Health Safety Net	1317 (4.4)	699 (5.8)	263 (8.4)	194 (2.9)	161 (2.1)
Index of neighborhood-level resources, mean (SD)^c^	−0.390 (0.989)	−0.496 (1.007)	−0.307 (0.939)	−0.343 (0.945)	−0.300 (1.003)
Comorbidities					
Any comorbidity	24 683 (83.1)	9837 (81.3)	2546 (81.0)	5280 (79.7)	7020 (89.6)
No. of comorbidities, mean (SD)	2.818 (2.673)	2.710 (2.668)	2.553 (2.434)	2.637 (2.652)	3.242 (2.743)
Most common comorbidities					
Depression	14 764 (49.7)	6119 (50.6)	1542 (49.0)	3082 (46.5)	4021 (51.3)
Hypertension, uncomplicated	10 262 (34.5)	3897 (32.2)	962 (30.6)	2220 (33.5)	3183 (40.6)
Obesity	9104 (30.6)	3652 (30.2)	908 (28.9)	1897 (28.6)	2647 (33.8)
Attributed clinician or clinic characteristics					
Schizophrenia caseload intensity					
High schizophrenia caseload intensity	7318 (24.6)	2984 (24.7)	785 (25.0)	1591 (24.0)	1958 (25.0)
Attributed clinician’s or clinic’s percentage of outpatient visits with any schizophrenia diagnosis, mean (SD)	6.2 (11.4)	6.2 (10.3)	6.6 (13.3)	12.3 (14.1)	0.9 (5.0)
Categories of attributed clinician’s or clinic’s percentage of outpatient visits with any schizophrenia diagnosis, %					
<1	12 449 (41.9)	4025 (33.3)	1210 (38.5)	95 (1.4)	7119 (90.8)
1-9	12 190 (41.0)	6077 (50.2)	1426 (45.4)	4117 (62.1)	570 (7.3)
10-19	2609 (8.8)	1107 (9.1)	237 (7.5)	1171 (17.7)	94 (1.2)
≥20	2465 (8.3)	895 (7.4)	271 (8.6)	1243 (18.8)	56 (0.7)

Abbreviations: NP, nurse practitioner; PA, physician assistant; PCP, primary care provider.

^a^ Differences across specialties are all statistically significant at *P* < .001, calculated using χ^2^ tests and 2-tailed, unpaired *t* tests as appropriate.

^b^ Unless otherwise indicated.

^c^ Index of neighborhood-level resources is based on the Structural Racism Effect Index^31^; a score of 0 indicates the national average, and lower numbers indicate higher resourced areas.

Many with schizophrenia did not receive guideline-concordant care (Table 2). In our sample, only 14 446 (62.3%) had high antipsychotic medication adherence. Moreover, although 21 421 (72.1%) received any psychosocial services, less than a quarter received psychotherapy at least once per quarter. Among those with antipsychotic medication use, 16 041 (71.4%) had diabetes screening. Inpatient hospitalization was common, with nearly one-fifth having at least one hospitalization with a principal diagnosis of schizophrenia. In addition, 1335 (4.5%) had high use of inpatient care.

**Table 2.** Receipt of Guideline-Concordant Care by Attributed Clinician or Clinic Specialty

Outcome	No. (%) of person-years^a^				
	Overall (N = 29 713)	Psychiatrist (n = 12 104)	Attributed clinician or clinic specialty		
			PA or NP with psychiatric specialty (n = 3144)	Mental health clinic (n = 6626)	PCP (n = 7839)
Primary outcomes					
Antipsychotic medication adherence^b^					
Medication adherence (PDC), adjusted for inpatient stays, mean (SD)	0.772 (0.256)	0.769 (0.257)	0.765 (0.261)	0.794 (0.239)	0.760 (0.268)
High medication adherence (PDC ≥0.8)	14 446 (62.3)	5569 (63.2)	1474 (62.8)	3557 (67.6)	3846 (63.5)
Any receipt of psychosocial services	21 421 (72.1)	8720 (72.0)	2512 (79.9)	5777 (87.2)	4412 (56.3)
Routine receipt of psychotherapy (at least once every quarter)	6840 (23.0)	3012 (24.9)	1009 (32.1)	2259 (34.1)	560 (7.1)
Any diabetes screening for those with any antipsychotic prescription^b^	16 041 (71.4)	6019 (68.3)	1508 (64.2)	3723 (70.8)	4791 (79.1)
High use of inpatient services for schizophrenia					
Any use of inpatient services with a principal diagnosis of schizophrenia	5690 (19.1)	2379 (19.7)	581 (18.5)	1320 (19.9)	1410 (18.0)
≥30 Inpatient days or ≥3 inpatient admissions with a principal diagnosis of schizophrenia	1335 (4.5)	544 (4.5)	142 (4.5)	327 (4.9)	322 (4.1)
Secondary outcomes					
Any use of antipsychotic medication^c^	22 483 (85.0)	8816 (83.1)	2349 (83.3)	5260 (91.2)	6058 (83.5)
High use of ED services					
Any use of ED services with a principal diagnosis of schizophrenia	7396 (24.9)	2827 (23.4)	706 (22.5)	1730 (26.1)	2133 (27.2)
≥4 ED visits with a principal diagnosis of schizophrenia	590 (2.0)	223 (1.8)	44 (1.4)	135 (2.0)	188 (2.4)

Abbreviations: ED, emergency department; NP, nurse practitioner; PA, physician assistant; PCP, primary care provider; PDC, proportion of days covered.

^a^ Unless otherwise indicated.

^b^ N = 22 483; psychiatrist n = 8816; PA or NP n = 2349; mental health clinic n = 5260; and PCP n = 6058. The denominator for this outcome includes person-years of antipsychotic medication use, excluding person-years insured by one insurer with unusual pharmacy claim submission pattern during 2017 to 2021.

^c^ N = 26 448; psychiatrist n = 10 606; PA or NP n = 2819; mental health clinic n = 5770; and PCP n = 7253. The denominator for this outcome includes all person-years, excluding person-years insured by one insurer with unusual pharmacy claim submission pattern during 2017 to 2021.

**Figure 1.**
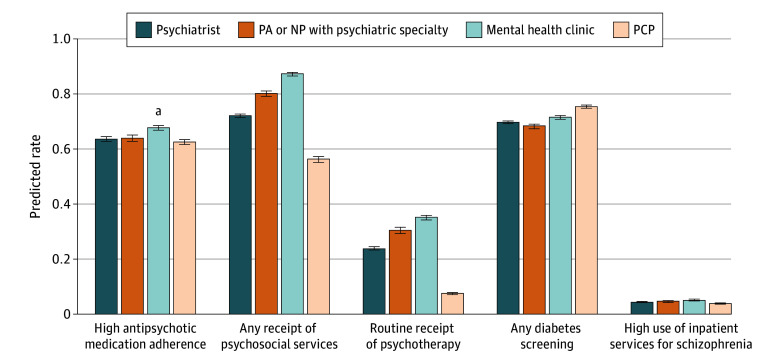
Regression-Adjusted Predicted Probabilities of Guideline-Concordant Care by Attributed Clinician or Clinic Specialty Predicted probabilities were calculated based on logistic regression models, including a categorical indicator of specialty, controlling for age category, sex, primary insurance type, comorbidities, and index of neighborhood-level resources at the 5-digit zip code level. Regression SEs were clustered at the 5-digit zip code level, and 95% CIs (error bars) for predicted probabilities were calculated using the Δ method. NP indicates nurse practitioner; PA, physician assistant; PCP, primary care provider. ^a^*P* < .05 for test comparison to psychiatrist.

Among the 4 clinician or clinic specialties (psychiatrists, PAs or NPs with a psychiatric specialty, mental health clinics, and PCPs), those whose attributed clinician or clinic was a PA or NP with a psychiatric specialty or a mental health clinic had higher probabilities of receiving psychosocial services (APP vs psychiatrist: 80.1% vs 72.1%, *P* < .001; mental health clinic vs psychiatrist: 87.1% vs 72.1%, *P* < .001) and routine psychotherapy (APP vs psychiatrist: 30.4% vs 24.0%, *P* < .001; mental health clinic vs psychiatrist: 35.2% vs 24.0%, *P* < .001) compared with those cared for by psychiatrists after regression adjustment (Figure 1; see secondary outcomes in eFigures 2 and 3, unadjusted results in eTable 4, and full results in eTable 5 in Supplement 1). Observations attributed to mental health clinics had higher adjusted probabilities of high antipsychotic medication adherence (4.05 percentage points [pp]; 95% CI, 1.71-6.39 pp) compared with those attributed to psychiatrists. In contrast, those attributed to PCPs were significantly less likely to receive psychosocial services (−15.85 pp; 95% CI, −18.40 to −13.30 pp) or routine psychotherapy than those under the care of psychiatrists (−16.42 pp; 95% CI, −17.80 to −15.05 pp). However, observations attributed to PCPs were more likely to receive diabetes screening while taking antipsychotic medication than those under the care of psychiatrists (5.66 pp; 95% CI, 4.02-7.30 pp). There was no statistically significant difference in high use of inpatient service across specialties.

**Figure 2.**
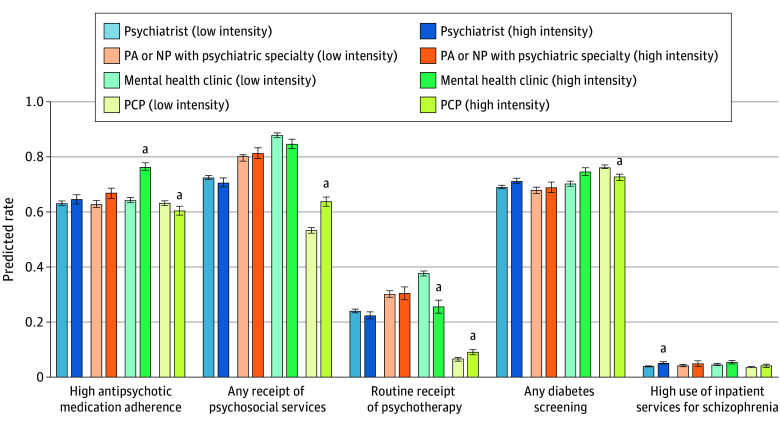
Regression-Adjusted Predicted Probabilities of Guideline-Concordant Care by High Schizophrenia Caseload Intensity Among Clinician or Clinic Within Each Specialty Predicted probabilities were calculated based on logistic regression models, including a categorical indicator of specialty, an indicator of high schizophrenia caseload intensity, and an interaction between these 2 variables, controlling for age category, sex, primary insurance type, comorbidities, and index of neighborhood-level resources at the 5-digit zip code level. Regression SEs were clustered at the 5-digit zip code level, and 95% CIs (error bars) for predicted probabilities are calculated using the delta method. NP indicates nurse practitioner; PA, physician assistant; PCP, primary care provider. ^a^*P* < .05 for statistical comparison of low vs high schizophrenia caseload intensity within each specialty.

The association of high schizophrenia caseload intensity differed by specialty (Figure 2; see secondary outcomes in eFigures 2 and 3, unadjusted results in eTable 6, and full results in eTable 7 in Supplement 1). However, greater caseload intensity was not consistently associated with improved outcomes within individual specialties. Overall, clinician or clinic schizophrenia caseload intensity had no significant association with outcomes for patients cared for by PAs or NPs with a psychiatric specialty. Among those attributed to mental health clinics, high schizophrenia caseload intensity was associated with high antipsychotic medication adherence (12.05 pp; 95% CI, 8.96-15.13 pp) and decreased routine receipt of psychotherapy (−12.03 pp; 95% CI, −17.20 to −6.86 pp). Among those attributed to a PCP, higher schizophrenia caseload intensity was associated with an increased probability of receiving any psychosocial services (10.37 pp; 95% CI, 7.25-13.49 pp) and routine psychotherapy (2.47 pp; 95% CI, 0.63-4.32 pp) but a decreased probability of high antipsychotic medication adherence (−2.79 pp; 95% CI, −5.82 to 0.24 pp; *P* = .04 for interaction) and diabetes screening while taking antipsychotic medication (−3.50 pp; 95% CI, −6.14 to −0.85 pp). Those attributed to psychiatrists with a high schizophrenia caseload intensity were more likely to exhibit high use of inpatient care (1.32 pp; 95% CI, 0.38-2.25 pp). Results from the sensitivity analysis eliminating 2020 and 2021 were similar in direction, magnitude, and significance.

## Discussion

In this statewide sample of insured adults with schizophrenia aged 18 to 64 years, we examined rates of guideline-concordant care by specialty and by schizophrenia caseload intensity within clinician or clinic specialty. Overall, we observed variations in service-related guideline-concordant care, including receipt of psychosocial services and routine psychotherapy, but minimal differences in other outcomes across those attributed to 4 clinician or clinic specialties. Specifically, those treated by psychiatric APPs and those seen at mental health clinics received guideline-concordant care at rates comparable or better to those treated by psychiatrists. Those attributed to PCPs were less likely to receive psychosocial services and routine psychotherapy but were more likely to undergo diabetes screening while taking antipsychotic medication. Among patients treated by psychiatrists and psychiatric APPs, there were minimal differences in guideline-concordant care rates by schizophrenia caseload intensity. In contrast, among those attributed to mental health clinics or PCPs, the association between schizophrenia caseload intensity and outcomes was mixed, showing both positive and negative associations.

The overall comparable rates of guideline-concordant care across clinician or clinic specialties, especially psychiatric APPs, are encouraging and suggest potential to expand access without compromising care quality. This finding aligns with prior research showing NPs across specialties deliver care quality similar to that of physicians,^38^ including in primary care settings for those with mental illness.^39^ Additionally, psychiatric NPs are generally associated with positive patient outcomes across care settings.^40^ Our findings also indicate that patients may benefit from PCPs with a high schizophrenia caseload intensity (vs other PCPs), particularly in improving adherence to service-related guideline-concordant care, such as psychosocial services and psychotherapy. PCPs play a particularly important role in rural areas, where they prescribe a substantial proportion of antipsychotics.^41^ However, fewer than half routinely refer patients for therapy.^42^ Finally, we observed no statistically significant differences in high use of inpatient care across specialties, suggesting broadly similar outcomes across groups. Caseload intensity is only associated with higher use of inpatient care among patients cared for by psychiatrists, with no statistically significant differences observed for other specialties.

### Limitations

Our study has limitations. First, our analysis is limited by the lack of detailed clinical information on schizophrenia severity, which raises the possibility of selection bias. Psychiatrists may treat patients with more severe symptoms or complex clinical needs, potentially complicating adherence to treatment guidelines. However, individuals with schizophrenia who have limited insight into their illness may be more likely to seek care from PCPs. Although there is evidence that PCPs more often refer people with schizophrenia to specialists,^43^ future research should examine differences in who is treated by each practitioner specialty. Although we observed no significant differences in comorbidities or high inpatient use (both an outcome and indicator of clinical severity) among clinician or clinic types, those attributed to PCPs tend to be older and more likely to be covered by Medicaid. We adjusted for these variables but cannot rule out unobservable differences in characteristics. Our attribution algorithm, based on outpatient visits within a calendar year, may not reflect the clinician or clinic responsible for most care during that year or during the illness and may not be generalizable to patients who do not receive outpatient care. Second, our data are limited to Massachusetts, a state with one of the lowest uninsured rates in the US.^44^ However, nationally, uninsurance rates among individuals with schizophrenia have remained low since the implementation of the Patient Protection and Affordable Care Act.^11^ Third, due to *Gobeille v. Liberty Mutual*,^45^ there is an underrepresentation of privately insured individuals in the data starting in 2016. The proportion of individuals with private insurance is broadly similar to previous studies^11,12^ of adults with schizophrenia. Fourth, the use of claims data limits what can reliably be captured relative to APA guidelines for patient-centered care.^46^ Claims data with multiple insurance types may not accurately reflect additional recommended services, such as peer support groups, supported employment and education, social skills training, and vocational rehabilitation. These data also cannot measure clinically indicated adverse effect management, although differences in this management may be reflected by medication adherence rates. Additionally, we examine routine receipt of psychotherapy, which may undercount out-of-pocket psychotherapy for those with private insurance.^47^ Although APA guidelines highlight the benefits of cognitive behavioral therapy for psychosis, claims data lack granularity to identify psychotherapy modality. Coordinated specialty care programs are indicated for those with early psychosis; as we focus on all adults with schizophrenia aged 18 to 64 years, future research should examine differences in referral and retention in coordinated specialty care by treating clinician or clinic type. We did not examine rates of clozapine use, a guideline-recommended treatment for people with treatment-resistant schizophrenia, but previous research^48,49^ documents very low rates of clozapine use across all practice settings.

## Conclusions

In this cross-sectional study of insured adults with schizophrenia aged 18 to 64 years, we found comparable rates of guideline-concordant care for patients treated by psychiatrists and those treated by APPs with psychiatry training or mental health clinics. Given the expanding role of APPs and PCPs, particularly in rural settings, and consistently low rates of guideline-concordant care across specialties, further research should examine strategies to improve care for people with schizophrenia.

## Notes

Supplement 1. **eTable 1. **Definitions of guideline-concordant care outcomes **eTable 2.** Definitions of provider specialty **eTable 3.** Descriptive statistics of people without mental health medication management or E&M visit **eTable 4. **Unadjusted logistic regression results of association of outcomes with specialty **eTable 5. **Adjusted full logistic regression results of association of outcomes with specialty **eTable 6. **Unadjusted logistic regression results of association of outcomes with specialty and high schizophrenia caseload intensity **eTable 7. **Adjusted full logistic regression results of association of outcomes with specialty and high schizophrenia caseload intensity **eFigure 1. **Study flow diagram **eFigure 2. **Regression-adjusted predicted rates of any antipsychotic medication use by specialty and low vs. high schizophrenia caseload intensity. **eFigure 3. **Regression-adjusted predicted rates of high use of emergency department (ED) services for schizophrenia by specialty and low vs. high schizophrenia caseload intensity.

Supplement 2.
Data Sharing Statement
