# Supplementary material for: Guideline-Concordant Care and Clinician and Clinic Characteristics for Patients With Schizophrenia
Source: JAMA Netw Open. 2025 Dec 26;8(12):e2549130. doi: 10.1001/jamanetworkopen.2025.49130 (PMC12743273; doi:10.1001/jamanetworkopen.2025.49130)
Supplement: Supplement 1. — eTable 1. Definitions of guideline-concordant care outcomes eTable 2. Definitions of provider specialty eTable 3. Descriptive statistics of people without mental health medication management or E&M visit eTable 4. Unadjusted logistic regression results of association of outcomes with specialty eTable 5. Adjusted full logistic regression results of association of outcomes with specialty eTable 6. Unadjusted logistic regression results of association of outcomes with specialty and high schizophrenia caseload intensity eTable 7. Adjusted full logistic regression results of association of outcomes with specialty and high schizophrenia caseload intensity eFigure 1. Study flow diagram eFigure 2. Regression-adjusted predicted rates of any antipsychotic medication use by specialty and low vs. high schizophrenia caseload intensity. eFigure 3. Regression-adjusted predicted rates of high use of emergency department (ED) services for schizophrenia by specialty and low vs. high schizophrenia caseload intensity. [file jamanetwopen-e2549130-s001.pdf]

Supplemental Online Content

Chen AY-A, Simon GE, Ericson KM, Zeber JE, Qian J, Geissler KH. Guideline-concordant care and practitioner characteristics for patients with schizophrenia. *JAMA Netw Open*. 2025;8(12):e2549130. doi:10.1001/jamanetworkopen.2025.49130

eTable 1. Definitions of guideline-concordant care outcomes

eTable 2. Definitions of provider specialty

eTable 3. Descriptive statistics of people without mental health medication management or E&M visit

eTable 4. Unadjusted logistic regression results of association of outcomes with specialty

eTable 5. Adjusted full logistic regression results of association of outcomes with specialty

eTable 6. Unadjusted logistic regression results of association of outcomes with specialty and high schizophrenia caseload intensity

eTable 7. Adjusted full logistic regression results of association of outcomes with specialty and high schizophrenia caseload intensity

eFigure 1. Study flow diagram

eFigure 2. Regression-adjusted predicted rates of any antipsychotic medication use by specialty and low vs. high schizophrenia caseload intensity.

eFigure 3. Regression-adjusted predicted rates of high use of emergency department (ED) services for schizophrenia by specialty and low vs. high schizophrenia caseload intensity.

This supplemental material has been provided by the authors to give readers additional information about their work.

© 2025 Chen AY-A et al. *JAMA Network Open.*

eTable 1: Definitions of guideline-concordant care outcomes

Outcome	Definition	Reference
high antipsychotic medication adherence	Proportion Days Covered (PDC)≥0.8. PDC is calculated using a SAS macro censoring inpatient stays and ED stays longer than one day with principal diagnosis of schizophrenia.	Chang 2015[a]
any receipt of psychosocial services	At least one claim with the following codes in a year:<br>• Psychosocial Visit CPT codes:<br>90832, 90833, 90834, 90836, 90837, 90838, 90839, 90840, 90845, 90846, 90847, 90849,<br>• 90853, 90875, 90876, 90880<br>Psychosocial Visit HCPCS codes:<br>G0176, G0177, G0409, G0410, G0411, H0004, H0032, H0035, H0036, H0037, H0038, H0039, H0040, H0045, H0046, H2000, H2001, H2011, H2012, H2013, H2014, H2017, H2018, H2019, H2020, S0201, S9480, S9484, S9485	Finnerty et al. (2016) and Texas Children's Health Plan HEDIS® Quick Reference[b,c]
routine receipt of psychotherapy	at least one claim with the following CPT codes every quarter: 90785, 90791, 90792, 90832, 90833, 90834,<br>90836, 90837, 90838, 90839, 90840, 90845, 90846, 90847, 90853, G0017, G0018<br>If a patient is hospitalized with a principal diagnosis of schizophrenia, assume that they received quarterly psychotherapy during inpatient stays.	CMS Billing and Coding: Psychiatric Diagnostic Evaluation and Psychotherapy Services[d]
Diabetes Screening for People with Schizophrenia Who Are Using Antipsychotic Medications (SSD)	Among those on antipsychotic medications, at least one claim with the following CPT codes in a year: 80047-8, 80050, 80053, 80069, 82947, 82950-1, 83036-7	NCQA and CareSource coding guide[e,f]
high use of inpatient services for schizophrenia	≥30 inpatient days or ≥3 inpatient admissions with a principal diagnosis of schizophrenia. Inpatient admissions are identified through:<br>• Site of service=21, 51<br>• Revenue code=0114, 0116, 0124, 0126, 0134, 0136, 0144, 0146, 0154, 0156, 0204	

© 2025 Chen AY-A et al. *JAMA Network Open.*

	• typeofclaimcleaned='002' AND typeofbillonfacilityclaims='11' AND serviceproviderentitytypequalifi='2'	
any use of antipsychotic medication	At least one prescription of antipsychotic drug in a year.	HEDIS NDC Lists from 2016-202[g]
high use of Emergency Department (ED) services for schizophrenia	≥4 ED visits with a principal diagnosis of schizophrenia. ED visits are identified through:<br>• site of service=23<br>• revenue code=0450, 0451, 0452, 0456, 0459, 0981<br>• CPT code=99281, 99282, 99283, 99284, 99285, 99291, 99292<br>• HCPCS code= G0383, G0384<br>• admissionsource = '7'	

Notes:
[a]: Chang A. A SAS Macro to Calculate the PDC Adjustment of Inpatient Stays. https://support.sas.com/resources/papers/proceedings15/3560-2015.pdf. Published 2015. Accessed March 7, 2025.
[b]: Finnerty M, Neese-Todd S, Pritam R, et al. Access to Psychosocial Services Prior to Starting Antipsychotic Treatment Among Medicaid-Insured Youth. *J Am Acad Child Adolesc Psychiat*ry. 2016;55(1):69-76.e63.
[c]: Texas Children's Health Plan. HEDIS® Quick Reference Use of First-Line Psychosocial Care for Children and Adolescents on Antipsychotics. https://www.texaschildrenshealthplan.org/sites/default/files/pdf/_HEDIS_One%20Sheeter_APP.pdf. Published 2021. Accessed Oct 24, 2024.
[d]: Centers for Medicare & Medicaid Services. Billing and Coding: Psychiatric Diagnostic Evaluation and Psychotherapy Services. https://www.cms.gov/medicare-coverage-database/view/article.aspx?articleid=57520&ver=37&bc=0. Published 2024. Accessed Oct 24, 2024.
[e]: National Committee for Quality Assurance. Diabetes and Cardiovascular Disease Screening and Monitoring for People With Schizophrenia or Bipolar Disorder (SSD, SMD, SMC). https://www.ncqa.org/hedis/measures/diabetes-and-cardiovascular-disease-screening-and-monitoring-for-people-with-schizophrenia-or-bipolar-disorder/. Published 2024. Accessed Oct 17, 2024.
[f]: CareSource. BEHAVIORAL HEALTH HEDIS®CODING GUIDE. https://www.caresource.com/documents/medicaid-in-bh-hedis-coding-guide/. Published 2021. Accessed Oct 17, 2024.
[g]: National Committee for Quality Assurance (NCQA). HEDIS Measures and Technical Resources. https://www.ncqa.org/hedis/measures/. Accessed July 21, 2025.

eTable 2: Definitions of provider specialty

Provider type	Primary taxonomy
Psychiatrist	2084A0401X, 2084B0040X, 2084F0202X, 2084N0600X, 2084P0005X, 2084P0015X, 2084P0800X, 2084P0802X, 2084P0804X, 2084P0805X
PA/NP with psychiatric specialty	363LP0808X, 364SP0807X, 364SP0808X, 364SP0809X, 364SP0810X, 364SP0812X, 364SP0813X
Mental health clinic (group NPI)	251S00000X, 261QM0801X
PCP	207Q00000X, 207QA0000X, 207QA0505X, 207R00000X, 207RA0000X, 208000000X, 2080A0000X, 2083P0500X, 2083P0901X, 208D00000X, 363A00000X, 363AM0700X, 363L00000X, 363LA2200X, 363LC1500X, 363LF0000X, 363LP0200X, 363LP2300X, 363LS0200X, 364SA2200X, 364SC1501X, 364SC2300X, 364SF0001X, 364SP0200X, 364SS0200X

© 2025 Chen AY-A et al. *JAMA Network Open.*

eTable 3: Descriptive statistics of people without mental health medication management or E&M visit

N (%) unless noted	**Person-years with outpatient visits (N=29,713)**	**Person-years without outpatient visits (N=10,946)**
**Guideline Concordant Care Outcomes**		
Antipsychotic medication adherence[a]		
Medication adherence (PDC), adjusted for inpatient stays [mean (standard deviation)]	0.772 (0.256)	0.682 (0.311)
High medication adherence (PDC≥0.8)	14,446 (62.3%)	3,940 (53.1%)
Any receipt of psychosocial services	21,421 (72.1%)	5,542 (50.6%)
Routine receipt of psychotherapy (at least once every quarter)	6,840 (23.0%)	689 (6.3%)
Any diabetes screening for those with any antipsychotic prescription[a]	16,041 (71.4%)	4,055 (54.7%)
High use of inpatient services for schizophrenia		
Any use of inpatient services with a principal diagnosis of schizophrenia	5,690 (19.1%)	2,731 (24.9%)
≥30 inpatient days or ≥3 inpatient admissions with a principal diagnosis of schizophrenia	1,335 (4.5%)	824 (7.5%)
Any use of antipsychotic medication[b]	22,483 (85.0%)	7,415 (72.0%)
High use of Emergency Department (ED) services		
Any use of ED services with a principal diagnosis of schizophrenia	7,396 (24.9%)	3,681 (33.6%)
≥4 ED visits with a principal diagnosis of schizophrenia	590 (2.0%)	261 (2.4%)
**Age**		
18-24	3,979 (13.4%)	1,793 (16.4%)
25-34	6,962 (23.4%)	2,943 (26.9%)
35-44	5,475 (18.4%)	1,999 (18.3%)
45-54	6,162 (20.7%)	1,926 (17.6%)
55-64	7,135 (24.0%)	2,285 (20.9%)
**Sex**		
Male	18,696 (62.9%)	7,716 (70.5%)
Female	11,017 (37.1%)	3,230 (29.5%)
**Insurance type**		
Health Safety Net	1,317 (4.4%)	224 (2.0%)
Medicaid	19,873 (66.9%)	8,337 (76.2%)
Private (including Marketplace)	5,393 (18.2%)	1,672 (15.3%)

© 2025 Chen AY-A et al. *JAMA Network Open.*

Medicare Advantage and integrated Medicare & Medicaid	3,130 (10.5%)	713 (6.5%)
**Index of neighborhood-level resources**[c] [mean (SD)]	-0.390 (0.989)	-0.158 (1.001)
**Comorbidities**		
Any comorbidity	24,683 (83.1%)	7,335 (67.0%)
Number of comorbidities [mean (SD)]	2.818 (2.673)	1.914 (2.328)
Most common comorbidities		
Depression	14,764 (49.7%)	4,269 (39.0%)
Hypertension, uncomplicated	10,262 (34.5%)	2,672 (24.4%)
Obesity	9,104 (30.6%)	1,496 (13.7%)

Note: Statistical significance for demographic comparisons across groups are all statistically significant with p<0.01, calculated using chi-squared tests and t-tests as appropriate.

[a]: denominator for this outcome excludes people insured under one insurer with unusual pharmacy claim submission patter during 2017-2021 and includes only person-years on antipsychotic medications. N=22,483 for person-years with outpatient visits. N=7,415 for person-years without outpatient visits.

[b]: denominator for this outcome excludes people insured under one insurer with unusual pharmacy claim submission patter during 2017-2021. N=26,448 for person-years with outpatient visits. N=10,299 for person-years without outpatient visits. Differences of variables across two groups are all statistically significant (P < .001 for chi-squared tests and t tests (index of neighborhood-level resources)).

[c]: Index of neighborhood-level resources is based on the Structural Racism Effect Index (Dyer et al); a score of 0 indicates the national average, and lower numbers indicate higher resourced areas.

© 2025 Chen AY-A et al. *JAMA Network Open.*

eTable 4: Unadjusted logistic regression results of association of outcomes with specialty

	1				2				3				4			
	**high antipsychotic medication adherence**				**any receipt of psychosocial services**				**routine receipt of psychotherapy**				**any diabetes screening**			
	**OR**	**95% CI**			**OR**	**95% CI**			**OR**	**95% CI**			**OR**	**95% CI**		
**Specialty (reference=psychiatrist)**																
PA/NP with psychiatric specialty	0.982	0.884	1.092		1.542	1.349	1.764	***	1.427	1.275	1.596	***	0.833	0.750	0.926	**
Mental health clinic	1.218	1.087	1.364	**	2.641	2.283	3.054	***	1.561	1.412	1.727	***	1.126	1.015	1.248	*
PCP	1.014	0.929	1.106		0.500	0.447	0.559	***	0.232	0.204	0.264	***	1.757	1.597	1.933	***

	5				A1				A2			
	**high use of inpatient services for schizophrenia**				**any use of antipsychotic medication**				**high use of ED services for schizophrenia**			
	**OR**	**95% CI**			**OR**	**95% CI**			**OR**	**95% CI**		
**Specialty (reference=psychiatrist)**												
PA/NP with psychiatric specialty	1.005	0.825	1.224		1.015	0.878	1.172		0.756	0.522	1.095	
Mental health clinic	1.103	0.939	1.296		2.094	1.807	2.427	***	1.108	0.884	1.388	
PCP	0.910	0.771	1.075		1.029	0.910	1.164		1.309	1.055	1.625	*

Note: PA=physician assistant, NP=nurse practitioner, PCP=primary care provider, ED=emergency department, OR=odds ratio, CI=confidence interval, ***P<0.001, **P<0.01, *P<0.05. The sample includes person-years with at least two non-hospitalization/ED visits or one hospitalization/ED visit where schizophrenia was listed as a principal or secondary diagnosis. Person-years without an attributed provider in a relevant specialty, based on mental health medication management or E&M visits, were excluded. The logistic regression models included a categorical indicator of specialty. No additional controls were included. Standard errors were clustered at the 5-digit zip code level.

© 2025 Chen AY-A et al. *JAMA Network Open.*

eTable 5: Adjusted full logistic regression results of association of outcomes with specialty

	1				2				3				4			
	**high antipsychotic medication adherence**				**any receipt of psychosocial services**				**routine receipt of psychotherapy**				**any diabetes screening**			
	**OR**	**95% CI**			**OR**	**95% CI**			**OR**	**95% CI**			**OR**	**95% CI**		
**Specialty (reference=psychiatrist)**																
PA/NP with psychiatric specialty	1.024	0.921	1.137		1.604	1.405	1.833	***	1.400	1.249	1.570	***	0.924	0.824	1.036	
Mental health clinic	1.209	1.083	1.350	**	2.757	2.365	3.214	***	1.775	1.614	1.953	***	1.104	0.999	1.220	
PCP	0.953	0.876	1.037		0.474	0.420	0.535	***	0.247	0.216	0.283	***	1.392	1.262	1.535	**

	5				A1				A2			
	**high use of inpatient services for schizophrenia**				**any use of antipsychotic medication**				**high use of ED services for schizophrenia**			
	**OR**	**95% CI**			**OR**	**95% CI**			**OR**	**95% CI**		
**Specialty (reference=psychiatrist)**												
PA/NP with psychiatric specialty	1.056	0.862	1.294		1.145	0.998	1.315		0.818	0.560	1.196	
Mental health clinic	1.157	0.985	1.360		1.521	1.322	1.750	***	0.973	0.773	1.224	
PCP	0.871	0.730	1.041		0.674	0.600	0.757	***	1.002	0.797	1.259	

Note: PA=physician assistant, NP=nurse practitioner, PCP=primary care provider, ED=emergency department, OR=odds ratio, CI=confidence interval, ***P<0.001, **P<0.01, *P<0.05. The sample includes person-years with at least two non-hospitalization/ED visits or one hospitalization/ED visit where schizophrenia was listed as a principal or secondary diagnosis. Person-years without an attributed provider in a relevant specialty, based on mental health medication management or E&M visits, were excluded. The logistic regression models included a categorical indicator of specialty, controlling for age category, sex, primary insurance type, comorbidities, and index of neighborhood-level resources at the 5-digit ZIP code level. Standard errors were clustered at the 5-digit zip code level.

© 2025 Chen AY-A et al. *JAMA Network Open.*

eTable 6: Unadjusted logistic regression results of specialty and high schizophrenia caseload intensity

	1				2				3				4			
	high antipsychotic medication adherence				any receipt of psychosocial services				routine receipt of psychotherapy				any diabetes screening			
	OR	95% CI			OR	95% CI			OR	95% CI			OR	95% CI		
**Specialty (reference= psychiatrist)**																
PA/NP with psychiatric specialty	0.937	0.833	1.053		1.524	1.308	1.776	***	1.419	1.256	1.604	***	0.866	0.764	0.983	*
Mental health clinic	1.055	0.948	1.174		2.762	2.324	3.283	***	1.744	1.549	1.964	***	1.029	0.922	1.149	
PCP	1.054	0.957	1.161		0.445	0.397	0.500	***	0.208	0.180	0.241	***	1.912	1.723	2.121	***
High schizophrenia caseload intensity	1.030	0.882	1.204		0.848	0.707	1.018		0.785	0.660	0.933	**	1.074	0.942	1.225	
High intensity# PA/NP with psychiatric specialty	1.199	0.976	1.474		1.049	0.789	1.395		1.029	0.776	1.363		0.857	0.674	1.090	
High intensity# Mental health clinic	1.793	1.417	2.268	***	0.848	0.605	1.189		0.583	0.418	0.814	**	1.425	1.152	1.761	**
High intensity# PCP	0.850	0.706	1.025		1.591	1.247	2.029	***	1.554	1.169	2.065	**	0.713	0.576	0.881	**

	5				A1				A2			
	high use of inpatient services for schizophrenia				any use of antipsychotic medication				high use of ED services for schizophrenia			
	OR	95% CI			OR	95% CI			OR	95% CI		
**Specialty (reference=psychiatrist)**												
PA/NP with psychiatric specialty	1.080	0.871	1.340		0.985	0.843	1.150		0.986	0.665	1.463	
Mental health clinic	1.115	0.920	1.352		1.833	1.571	2.139	***	1.241	0.949	1.624	
PCP	0.978	0.815	1.174		1.082	0.953	1.228		1.543	1.183	2.011	**
High schizophrenia caseload intensity	1.375	1.125	1.681	**	1.191	1.004	1.413	*	1.661	1.235	2.236	**
High intensity# PA/NP with psychiatric specialty	0.772	0.493	1.209		1.123	0.787	1.604		0.340	0.146	0.790	*
High intensity# Mental health clinic	0.971	0.693	1.362		2.037	1.447	2.868	***	0.695	0.429	1.125	
High intensity# PCP	0.773	0.558	1.070		0.803	0.645	1.000	*	0.568	0.359	0.900	*

© 2025 Chen AY-A et al. *JAMA Network Open.*

Note: PA=physician assistant, NP=nurse practitioner, PCP=primary care provider, ED=emergency department, OR=odds ratio, CI=confidence interval, ***P<0.001, **P<0.01, *P<0.05.

The sample includes person-years with at least two non-hospitalization/ED visits or one hospitalization/ED visit where schizophrenia was listed as a principal or secondary diagnosis. Person-years without an attributed provider in a relevant specialty, based on mental health medication management or E&M visits, were excluded. The logistic regression models interacted specialty with high schizophrenia caseload intensity. No additional controls were included. Standard errors were clustered at the 5-digit zip code level.

© 2025 Chen AY-A et al. *JAMA Network Open.*

eTable 7: Adjusted full logistic regression results of specialty and high schizophrenia caseload intensity

	1				2				3				4			
	high antipsychotic medication adherence				any receipt of psychosocial services				routine receipt of psychotherapy				any diabetes screening			
	OR	95% CI			OR	95% CI			OR	95% CI			OR	95% CI		
**Specialty (reference=psychiatrist)**																
PA/NP with psychiatric specialty	0.989	0.876	1.115		1.522	1.304	1.777	***	1.374	1.215	1.554	***	0.936	0.817	1.073	
Mental health clinic	1.058	0.949	1.179		2.895	2.417	3.468	***	1.960	1.753	2.192	***	1.067	0.953	1.195	
PCP	1.004	0.910	1.108		0.411	0.363	0.465	***	0.222	0.190	0.259	***	1.515	1.357	1.691	***
High schizophrenia caseload intensity	1.070	0.918	1.247		0.913	0.759	1.099		0.906	0.778	1.055		1.129	0.996	1.279	
High intensity# PA/NP with psychiatric specialty	1.120	0.904	1.387		1.226	0.928	1.620		1.119	0.843	1.486		0.933	0.717	1.215	
High intensity# mental health clinic	1.730	1.360	2.201	***	0.828	0.586	1.171		0.613	0.446	0.841	**	1.141	0.917	1.419	
High intensity# PCP	0.824	0.684	0.994	*	1.740	1.341	2.258	***	1.548	1.165	2.057	**	0.717	0.591	0.869	**

	5				A1				A2			
	high use of inpatient services for schizophrenia				any use of antipsychotic medication				high use of ED services for schizophrenia			
	OR	95% CI			OR	95% CI			OR	95% CI		
**Specialty (reference=psychiatrist)**												
PA/NP with psychiatric specialty	1.099	0.878	1.375		1.087	0.933	1.267		1.060	0.707	1.591	
Mental health center	1.196	0.987	1.449		1.399	1.202	1.628	***	1.127	0.856	1.484	
PCP	0.914	0.755	1.106		0.695	0.613	0.787	***	1.161	0.881	1.529	
High schizophrenia caseload intensity	1.352	1.107	1.650	**	1.133	0.961	1.335		1.806	1.299	2.511	***
High intensity# PA/NP with psychiatry specialty	0.873	0.537	1.419		1.221	0.865	1.724		0.351	0.149	0.829	*
High intensity# Mental health clinic	0.872	0.622	1.224		1.623	1.166	2.258	**	0.584	0.354	0.965	*
High intensity# PCP	0.863	0.621	1.199		0.863	0.621	1.199		0.611	0.378	0.988	*

© 2025 Chen AY-A et al. *JAMA Network Open.*

Note: PA=physician assistant, NP=nurse practitioner, PCP=primary care provider, ED=emergency department, OR=odds ratio, CI=confidence interval, ***P<0.001, **P<0.01, *P<0.05.

The sample includes person-years with at least two non-hospitalization/ED visits or one hospitalization/ED visit where schizophrenia was listed as a principal or secondary diagnosis. Person-years without an attributed provider in a relevant specialty, based on mental health medication management or E&M visits, were excluded. The logistic regression models interacted specialty with high schizophrenia caseload intensity, controlling for age category, sex, primary insurance type, comorbidities, and index of neighborhood-level resources at the 5-digit ZIP code level. Standard errors were clustered at the 5-digit zip code level.

© 2025 Chen AY-A et al. *JAMA Network Open.*

eFigure 1: Study flow diagram

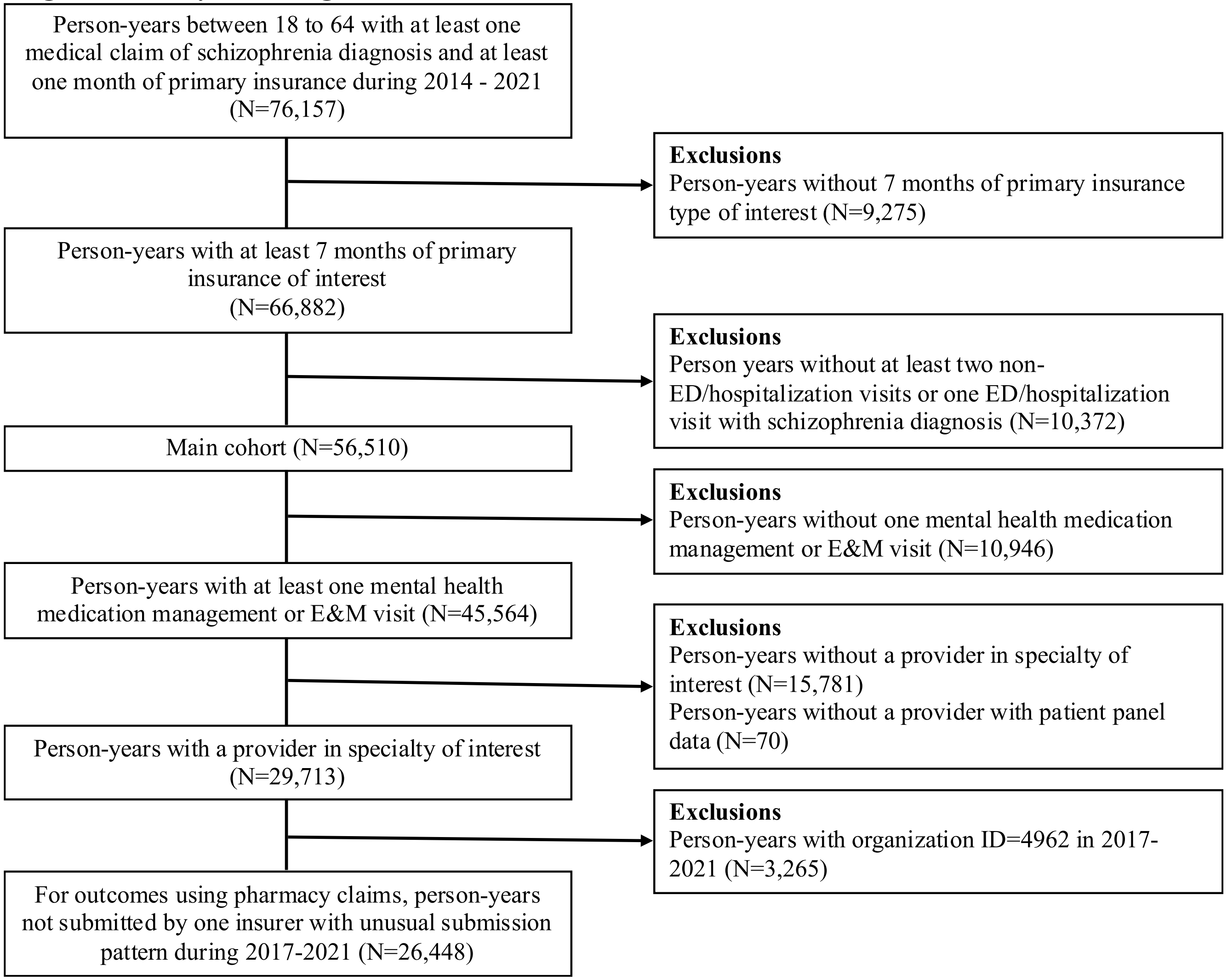

Note: Primary analytic sample is person-years with a provider in specialty of interest. Last box is used for outcomes of any antipsychotic medication and diabetes screening for those on any antipsychotic as noted in the text.

© 2025 Chen AY-A et al. *JAMA Network Open.*

eFigure 2: Regression-adjusted predicted probabilities of any antipsychotic medication use by specialty and low vs. high schizophrenia caseload intensity.

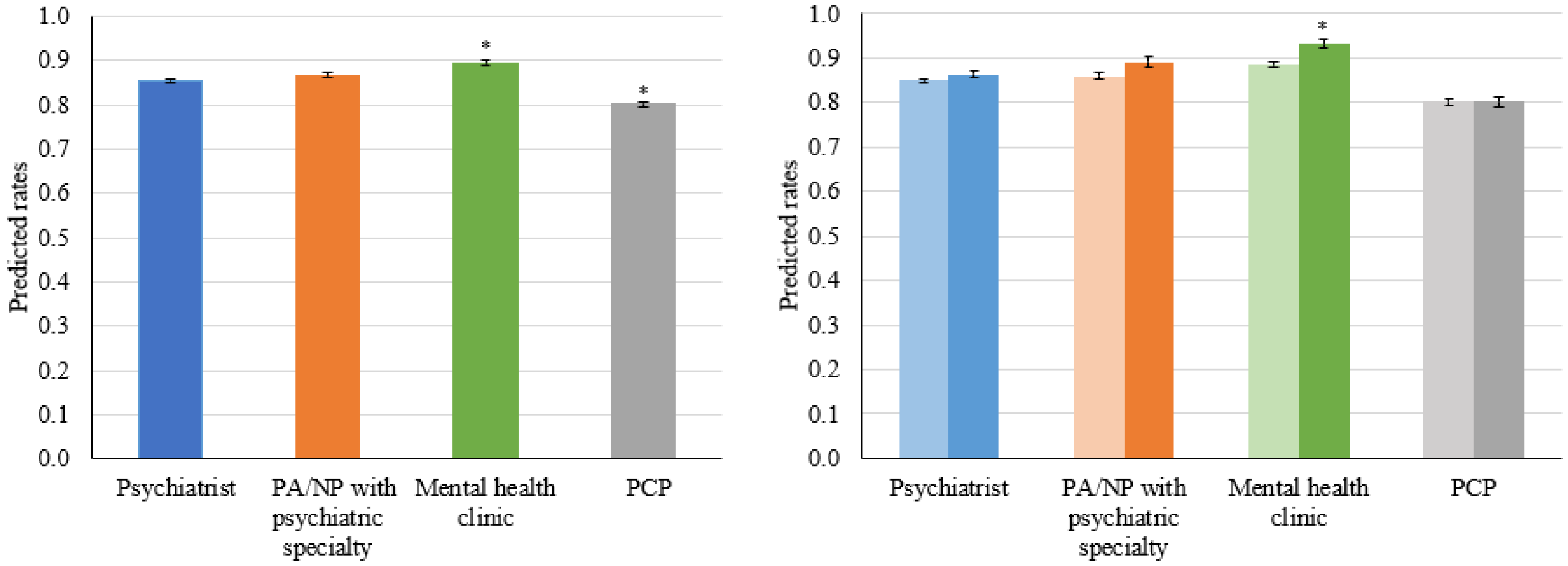

Note: PA=physician assistant, NP=nurse practitioner, PCP=primary care provider. Test result for the left figure is testing against psychiatrist, while the right figure is testing low vs. high schizophrenia caseload intensity in each specialty. *P<0.05. For right panel, darker color indicates high intensity within each specialty.

Predicted probabilities for left figure calculated based on logistic regression models including a categorical indicator of specialty, controlling for age category, sex, primary insurance type, comorbidities, and index of neighborhood-level resources at the 5-digit ZIP code level. Predicted probabilities for right figure calculated based on logistic regression models including a categorical indicator of specialty, an indicator of high schizophrenia caseload intensity, and an interaction between these two variables, controlling for age category, sex, primary insurance type, comorbidities, and index of neighborhood-level resources at the 5-digit ZIP code level. Regression standard errors were clustered at the 5-digit zip code level, and confidence intervals for predicted probabilities are calculated using the delta method.

© 2025 Chen AY-A et al. *JAMA Network Open.*

eFigure 3: Regression-adjusted predicted probabilities of high use of emergency department (ED) services for schizophrenia by specialty and low vs. high schizophrenia caseload intensity.

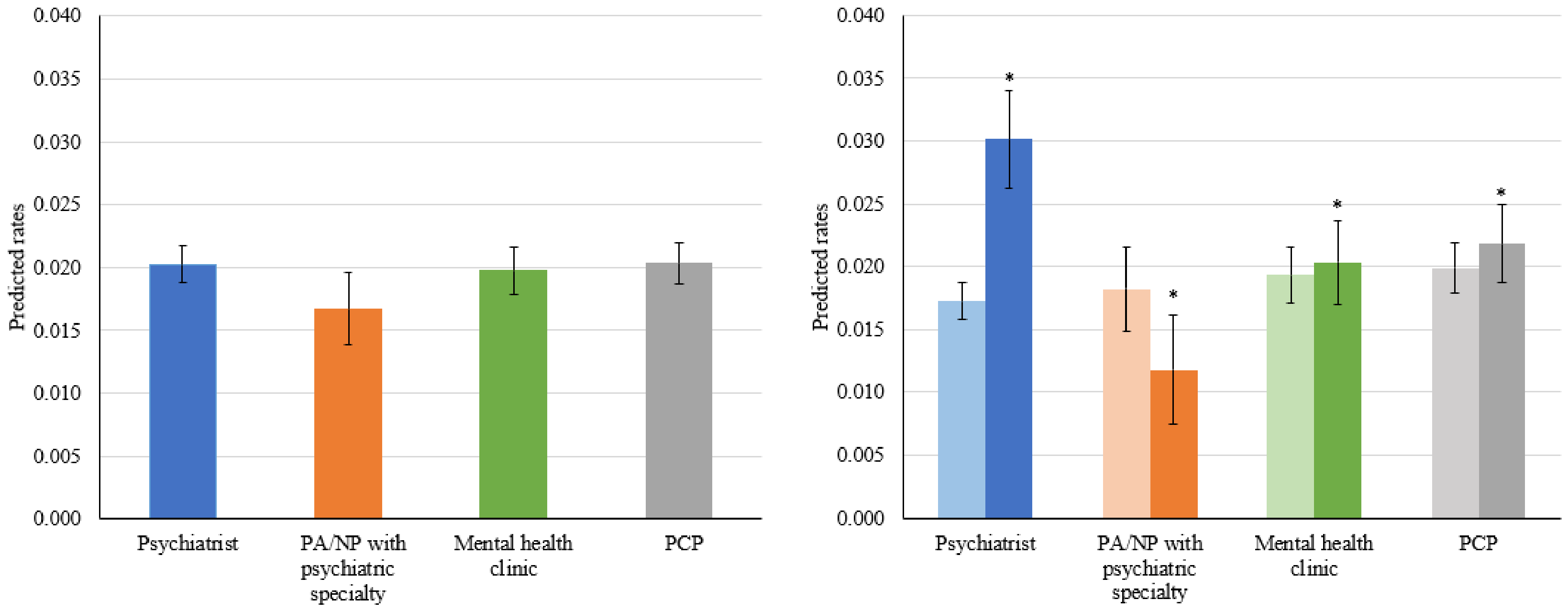

Note: PA=physician assistant, NP=nurse practitioner, PCP=primary care provider. Test result for the left figure is testing against psychiatrist, while the right figure is testing low vs. high schizophrenia caseload intensity in each specialty. *P<0.05. For right panel, darker color indicates high intensity within each specialty.

Predicted probabilities for left figure calculated based on logistic regression models including a categorical indicator of specialty, controlling for age category, sex, primary insurance type, comorbidities, and index of neighborhood-level resources at the 5-digit ZIP code level. Predicted probabilities for right figure calculated based on logistic regression models including a categorical indicator of specialty, an indicator of high schizophrenia caseload intensity, and an interaction between these two variables, controlling for age category, sex, primary insurance type, comorbidities, and index of neighborhood-level resources at the 5-digit ZIP code level. Regression standard errors were clustered at the 5-digit zip code level, and confidence intervals for predicted probabilities are calculated using the delta method.

© 2025 Chen AY-A et al. *JAMA Network Open.*
