# Supplementary material for: Guideline-Concordant Care and Clinician and Clinic Characteristics for Patients With Schizophrenia
Source: JAMA Netw Open. 2025 Dec 26;8(12):e2549130. doi: 10.1001/jamanetworkopen.2025.49130 (PMC12743273; doi:10.1001/jamanetworkopen.2025.49130)
Supplement: Supplement 2. — Data Sharing Statement [file jamanetwopen-e2549130-s002.pdf]

Data Sharing Statement

Chen. Guideline-Concordant Care and Clinician and Clinic Characteristics for Patients With Schizophrenia. *JAMA Netw Open*. Published December 26, 2025.
doi:10.1001/jamanetworkopen.2025.49130

Data
Data available: No

Additional Information
Explanation for why data not available: The data used for this analysis was provided by the Center for Health Information and Analysis (CHIA). Data sharing is restricted by terms in the Data Use Agreement.
